# Beyond the Check-Up: How Well-Child Exams, Pediatric Specialists, and Provider Recommendations Can Close HPV Vaccine Gaps for Chinese American Teens

**DOI:** 10.21203/rs.3.rs-7221867/v1

**Published:** 2025-08-27

**Authors:** Lin Zhu, Xinrui Li, Elaine Zhiqing Liu, Philip T. Siu, Shumenghui Zhai, Chun Pan, Nikki Cao, Grace X. Ma

**Affiliations:** Temple University; Vanderbilt University; Temple University; Esperanza Health Center; Pacific Lutheran University; Hunter College; Temple University; Temple University

**Keywords:** human papillomavirus vaccine, Chinese American, multilevel factors, disaggregated data, adolescents

## Abstract

**Purpose:**

This study aimed to identify provider- and practice-level factors influencing human papillomavirus (HPV) vaccine uptake among Chinese American (CA) adolescents. Despite increasing public health efforts, HPV vaccination rates in this population remain suboptimal, and knowledge on the link between provider/practice characteristics and vaccine uptake is limited.

**Methods:**

We analyzed data from 1,272 CA adolescents (aged 13–17) using the National Immunization SurveyTeen (2015–2019) datasets. Key measures included HPV vaccine initiation and completion (based on proxy reporting and verification), accompanied by various multilevel factors: provider-level (recommendation, specialty), practice-level (facility type, vaccine acquisition, well-child exam), parentlevel (mother’s education, marital status), and adolescent-level characteristics. Weighted descriptive statistics and stepwise multivariate logistic regression were used to examine associations.

**Results:**

Overall, 71.22% of CA adolescents initiated at least one HPV vaccine dose, while 48.14% completed the regimen. Provider's recommendation (initiation OR = 21.50, completion OR = 8.12), having a pediatrician (initiation OR = 5.37, completion OR = 3.20), and receiving the 11–12-year-old well-child exams significantly predict both initiation and completion. Regional disparities were observed, with adolescents in the Northeast and West showing higher completion rates than those in the South. Unexpectedly, adolescents not enrolled in school and those with mothers who had less than 12 years of education or were unmarried showed higher completion rates.

**Conclusion:**

Provider recommendations and pediatric care are crucial for improving HPV vaccination rates among CA adolescents. Addressing regional disparities and implementing targeted interventions, including enhanced provider training focused on culturally sensitive communication, are essential to dismantle systemic barriers and improve comprehensive vaccination coverage in this underserved population.

## Introduction

Sexually transmitted infections (STIs) pose a significant public health challenge, with rates steadily increasing since 2019.^1^ Human papillomavirus (HPV) is the most common form of sexually transmitted disease in the United States, affecting 42 million people.^2^ HPV is a leading cause of six cancers—anal, cervical, oropharyngeal, penile, vaginal, and vulvar—causing approximately 36,000 cases annually in the United States.^3^ Among these, cervical cancer has historically been the most common and a leading cause of cancer deaths in women. Importantly, HPV-related cancers are largely preventable. Current vaccines show over 90% efficacy against HPV-associated cancers and genital warts.^3^ To optimize the preventive effects, the Advisory Committee on Immunization Practices (ACIP) recommends routine vaccination at ages 11–12, before most sexual exposure occurs.^4^

Asian Americans are one of the fastest-growing demographic groups, currently comprising 7% of the population (twenty-four million) and projected to quadruple by 2060.^5^ Although Asian Americans comprise over 20 distinct ethnicities with diverse cultural, linguistic, and social backgrounds, research often treats them as a homogeneous group, masking subgroup disparities.^6–8^ These disparities remain understudied.^9^ Chinese Americans (CA) are the largest Asian subgroup (21.66%), yet recent research on their health disparities is limited.^10^ CAs are disproportionately affected by cervical cancer and several other HPV-related cancers.^11–13^ However, our understanding of their HPV vaccine uptake behaviors is limited.

According to the National Health Interview Survey from 2006 to 2018, less than half of the CA population completed their HPV, well below the Healthy People 2030 goal of 80% HPV vaccine uptake initiated by the U.S. Department of Health and Human Services.^14,15^ Developing targeted interventions to address these disparities requires an understanding of the multilevel factors affecting HPV vaccine uptake. Prior studies have primarily examined parent-level characteristics (e.g., income, education, and health insurance status), which significantly influence HPV vaccine uptake among CA adolescents.^16,17^ Additional determinants such as HPV vaccine knowledge, perceived safety, and disease susceptibility have also been identified as crucial factors.^18–20^ Beyond individual and parental influences, research has also suggested associations between vaccination rates and provider recommendations, practice-level factors, and structural barriers (e.g., lack of access) in the general population.^21–23^ The importance of provider- and practice-level characteristics in vaccine uptake remain understudied in the CA community.

This study aims to address this gap by examining disaggregated data on CA adolescents. To our knowledge, this will be the first to utilize a disaggregated nationally representative sample of CA adolescents to investigate the combined influence of multilevel factors, particularly provider- and practice-level characteristics, on HPV vaccine uptake. By analyzing these factors, this study will provide comprehensive insights to inform targeted public health interventions for improving vaccination rates in this population.

## Methods

### Data

This study included data from the National Immunization Survey-Teen (NIS-Teen) collected between 2015–2019. The NIS-teen is an ongoing survey supported by the CDC and conducted by the National Opinion Research Center (NORC). NIS-Teen collects demographic information and HPV vaccination status from parents or guardians of adolescents aged 13–17 through household surveys. The household surveys collect data through telephone interviews with parents or guardians in all 50 states, the District of Columbia, and some U.S. territories. Cell phone numbers are randomly selected and called to enroll one or more age-eligible teens from the household. During the call, the parents and guardians of eligible teens are asked for the names of their children’s vaccination providers and permission to contact them.

Having received verbal consent from parents or guardians, a questionnaire is sent via mail to the adolescents’ immunization providers to request information on the types of vaccinations, number of doses, dates of administration, and other administrative data about the health care facility.^24^ For simplicity, we’ll refer to the parent or guardian interviewed as the adolescent’s parent. Population-level weights are used to account for the multi-level sampling design used and the methods and weighting procedures for NIS-Teen were described previously.^25^

### Measures

#### HPV vaccine initiation and completion.

HPV vaccine uptake status was determined using data from the provider survey. We defined HPV vaccination initiation as a binary variable (yes/no), with “yes” indicating that the adolescent had received at least one dose of the HPV vaccine, according to the provider-reported number of HPV vaccine doses administered by the time of the interview. HPV vaccination completion was also defined as a binary variable (yes/no), based on the age at initiation and the number of doses received. Specifically, completion was defined as receiving two doses if the vaccine series was initiated before the age of 15 or three doses if the series was initiated at or after the age of 15, in accordance with the recommended HPV vaccination schedule.^2^

#### Provider- and practice-level factors.

This study examined two provider-level factors and three practice-level factors. The provider-level factors were: (1) whether the provider recommended the HPV vaccine to adolescents (binary variable: yes/no) and (2) the provider's specialty, which was categorized into six groups: pediatrics, family practice, general practice, internal medicine, obstetrics/gynecology (OB/GYN), and other. The three practice-level factors were as follows: Firstly, the types of healthcare facilities were categorized into all public, all private, all hospital facilities, all specialized clinics (e.g., STD/school/teen clinics), mixed, and others. Secondly, vaccine acquisition source was determined by whether the practices ordered HPV vaccines from the state/local health department through the Vaccine for Children (VFC) program (i.e., yes/no). Lastly, practices reported whether they conducted the 11–12-year-old well child exam or check-up for adolescents (i.e., yes/no).

#### Parent-level factors.

We examined two sociodemographic characteristics of the parents, obtained from the household survey. The first characteristic was the mother's educational level, which was categorized into four groups: less than 12 years of education, 12 years of education, more than 12 years of education but not a college graduate, and college graduate. The second characteristic was the mother's marital status, which was categorized into two groups: currently married and not currently married.

#### Adolescent-level factors.

We also examined seven characteristics of adolescents from the household survey. Sex was categorized as male or female. The Census region of residence was categorized into four groups: Northeast, Midwest, South, and West. Current grade of the adolescents was categorized into three groups: 6th to 8th grade, 9th to 12th grade, and high school (HS)/general educational development (GED)/not in school. Health insurance status was categorized into four groups: private insurance only, any Medicaid, other insurance, and uninsured. Household poverty level was categorized into three groups: above poverty level and >$75k, above poverty level <= $75k, and below poverty level. Nativity status was categorized into two groups: whether the adolescent was born in the US or not.

### Statistical Analysis

This study included 1,272 Chinese American adolescents who had sufficient provider data to determine vaccination status. To ensure that the sampled individuals were representative of the U.S. population demographics, we followed the instructions provided in the NIS-Teen User's Guide and applied the appropriate weights and stratum variables.^24^ This adjustment accounted for non-response, unresolved telephone numbers, missing provider data, and the complex survey design. When calculating average estimates over multiple years, we combined the NIS-Teen data from the relevant years and divided the annual weights by the number of years included in the analysis, as required by the NIS-Teen User's Guide for multi-year data amalgamation.^24^ Weighted analyses were conducted for all statistical tests. Weighted descriptive statistics, including percentages and 95% confidence intervals (CIs), were presented for various adolescent-, parent-, provider-, and practice-level characteristics of the Chinese American sample. For comparison purposes, descriptive statistics for the aggregate Asian sample were also provided. Stepwise multivariate logistic regression was employed to examine the multilevel factors associated with HPV vaccine initiation and completion, and the odds ratios (ORs) with 95% CIs were reported. All statistical analyses were performed using Stata version 16, with the *svy* command used to apply weighting and the *subpop* option used for subpopulation analyses.^26^ Statistical significance was determined at a p-value < 0.05. Figures are generated in RStudio (version 4.4.2) with packages dplyr (version 1.1.4), tidyr (version 1.3.1), ggplot2 (version 3.5.1), ggtext (version 0.1.2), and scales (version 1.3.0).^27^

## Results

The weighted socioeconomic and immigration-related characteristics of the adolescents and parents of the CA adolescents and the aggregate Asian American adolescent sample are presented in Table 1. Among the study sample, the mean age was 14.96 years and consisted of 37.49% boys and 62.51% girls. 21.72% of the adolescents had a family income of $75K or less, with 3.24% without any health insurance. Moreover, 12.76% of CA adolescents were born outside of the US. With regards to parental socioeconomic status, 18.24% of the CA adolescents reported that their mothers were not married at the time of the survey, and about three quarters (75.43%) had mothers with a college degree. The CA sample varied significantly from the aggregate AA sample in several characteristics. The proportion of CA adolescents reside in West (41.82%) was higher than the AA adolescents (36.19%). A higher percentage of CA adolescents had private insurance coverage (75.47%) compared to AA adolescents (65.09%). CA adolescents also had a higher proportion of household incomes above $75,000 and mothers who were college graduates compared to AA adolescents. Conversely, AA adolescents had a higher proportion of Medicaid coverage than CA adolescents.

Table 1 also presents the weighted provider- and practice-level characteristics for the CA sample and the aggregated Asian American sample. Among CA adolescents, 81.9% received a recommendation for the HPV vaccine from their providers. Over half 62.62% reported hospitals as the only types of facilities where their providers were located. The majority (75.20%) of the CA adolescents had a pediatrician, 28.80% had a family practitioner, while 20.12% had an internalist. About four out of five (81.47%) in the CA sample had a provider who ordered vaccines from state or local health departments. In addition, 95.94% of the CA adolescents reported receiving the 11–12-year-old well child exam from their providers. We noted significant variations between the CA and aggregate AA sample on several factors, including providers' recommendations for the HPV vaccine and whether the adolescents received an 11–12-year-old well child exam. Specifically, CA adolescents were more likely to receive a recommendation for the HPV vaccine and to have had an 11–12-year-old well child exam compared to the aggregated AA adolescents.

**Figure 1.**
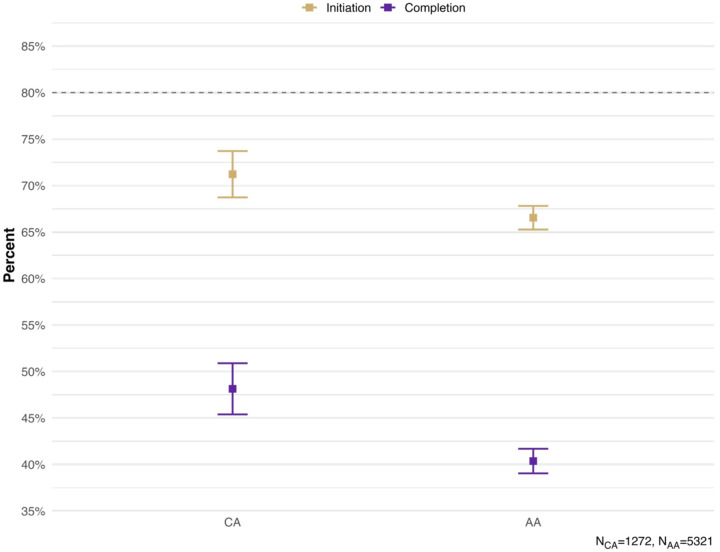
HPV Vaccination Initiation and Completion Rate in CA and Aggregate AA Adolescents, NIS-Teen 2015–2019.

Figure 1 present the weighted HPV vaccination initiation and completion rates, as recorded in the providers’ records. Based on the provider reports, only about two thirds (71.22%) of the CA adolescents received at least one shot, while about half (51.86%) did not complete their HPV vaccine regimen. Vaccine uptake was even lower according to household- reported data with only half (51.52%) initiating the vaccine and one in five (21.85%) completing the HPV vaccine regimen (results not included). In addition, “not receiving a recommendation from the provider” was cited by 25.17% of the unvaccinated CA adolescents as a reason for not receiving any HPV vaccines. This was closed 24.19% rate in the aggregated AA adolescents. Other reasons were examined, but the results were not presented because of low case counts, in compliance with the NIS-Teen data release policy.

Table 2 presents the results of the multivariate logistic regression on HPV vaccination initiation. We found that receiving a physician's recommendation for the HPV vaccine (OR=21.50, 95%CI=9.48–48.74) was significantly associated with a higher likelihood of initiating the HPV vaccine regimen. Having a pediatrician as a healthcare provider (OR=5.37, 95% CI=2.04–14.18) was a significant predictor of HPV vaccination initiation. Additionally, adolescents who received an 11–12-year-old well child exam were more likely to initiate HPV vaccination, although this association was only marginally significant (OR=4.12, 95% CI=0.94–17.97). In addition, our results showed that CA adolescents living in the Northeast was more likely than those living in the South to initiate HPV vaccination (OR=5.13, 95%CI=2.15–12.22).

Table 3 presents the association of various factors with HPV vaccination completion in CA adolescents. Having a provider recommendation (OR = 8.12, 95% CI = 3.54 – 18.60) and having a pediatrician (OR = 3.20, 95% CI = 1.24 – 8.22) were both significant predictors of completing the HPV vaccine regimen. Additionally, living in the West (OR = 2.84, 95% CI = 1.28–6.32) or Northeast (OR = 2.24, 95% CI = 1.18–4.25) was significantly associated with higher HPV vaccination completion compared with adolescents living in the South. Adolescents with mothers who are not currently married have a higher likelihood of HPV vaccination completion (OR = 2.34, 95% CI = 1.15 – 4.75). Surprisingly, CA adolescents in 6th-8th grade (OR=0.0021, 95% CI=0.00–0.26) and those in 9th-12th grade (OR=0.0032, 95% CI=0.00–0.39) were significantly less likely to complete the HPV vaccine regimen compared to those who do not currently enroll in school. Moreover, having mothers with less than 12 years of education was significantly associated with higher HPV completion likelihood compared to having mothers of college graduates (OR = 5.06, 95% CI = 1.53 – 16.73).

## Discussion

To the best of our knowledge, this study is one of the first to apply a nationally representative sample to examine HPV vaccine uptake and to investigate practice- and provider-level factors influencing HPV vaccine uptake among CA adolescents. The CA is the largest Asian American subgroup, accounting for approximately 22% of the Asian Americans.^28,29^ As a result of an aggregated multiple-year cross-sectional design, our findings captured the cancer prevention trend in CAs and support data-driven public health policies. Although CA adolescents show slightly higher HPV vaccine uptake than in aggregated Asian peers (71% vs. 67% initiation; 48% vs. 40% completion), these rates remain below the 80% benchmark set by Healthy People 2030.^15^ Additionally, our multilevel model revealed significant associations between provider, parent, and adolescent-level factors and HPV vaccine uptake.

Provider recommendations emerged as the strongest predictor of both initiating and completing the HPV vaccine series. This aligns with previous studies and reinforces the need for provider recommendations on HPV vaccination.^30–32^ Notably, while 95.94% of CA adolescents attended well-child exams, nearly 20% did not receive a recommendation, and 25% cited this as a primary barrier. This disconnect suggests that providers may not consistently prioritize HPV vaccine discussions during routine visits, even for this high-risk population.^30,33–35^ Our findings also highlight the need for provider training that emphasizes culturally attuned vaccine communications.

Specialty also influenced vaccine uptake. Recommendations from pediatricians (and, to a lesser extent, internists) were significantly associated with higher uptake. In contrast, providers in family medicine, OB/GYN, and general practice may be less likely to promote HPV vaccination due to training gaps or role differences.^33,36–39^ To address this disparity, mandatory HPV vaccine competency training for all primary care specialties through continuing medical education (CME) programs,^40^ electronic health record (EHR) prompts specifically for family medicine providers during adolescent visits,^41^ and cross-specialty reimbursement incentives are potential strategies to encourage broader HPV vaccine administration.^42^

Regional differences in healthcare access and resource allocation also emerged. Adolescents in the South (and the Midwest) had significantly lower HPV vaccine uptake compared to the Northeast and West. These differences may reflect varying policies, provider availabilities, and socioeconomic factors.^43–46^ Although the Vaccines for Children (VFC) program did not reach significance in our analysis, it remains a potentially valuable tool for low-income CA families. Future studies should evaluate how state-level infrastructure and outreach contribute to regional gaps.

Unexpectedly, we observed that CA adolescents not enrolled in school had higher odds of completing HPV vaccination compared to those attending traditional schools. Furthermore, CA mothers with less than 12 years of education or those who were currently unmarried had significantly higher odds of their children completing the HPV vaccination series. These findings, which differed from general population trends, may reflect increased engagement with public health services or differing levels of trust in healthcare messaging.^47–49^ Future research is needed to explore these dynamics among CA subgroups.

Our study has several limitations. Firstly, the cross-sectional nature of the data restricted the identification of health determinants. Longitudinal studies are needed to understand multilevel determinants of vaccine uptake. Secondly, our data only included HPV uptake information from provider-reported data, which may underrepresent vaccine access from community pharmacies, which play a critical role in Asian American communities.^49–51^ Building on the widespread accessibility of community pharmacies, particularly in underpriviledged communities and medically underserved regions, researchers have recognized their potential as strategic venues for improving HPV vaccination rates.^52,53^ Future studies should investigate pharmacy-based and culturally responsive interventions.

The findings of this study carry heightened importance for population health and cancer prevention, particularly in the post-pandemic landscape. Starting in 2020, HPV vaccination initiation rates experienced their first stagnation since 2013.^54^ This trend is likely attributable to increased vaccine hesitancy and a decrease in well-child appointment attendance during and after the pandemic. Providers across various specialties consistently recommend the HPV vaccine. Moreover, state and national policies and programs need to enhance their overall quality and adaptability to effectively dismantle the systemic barriers that low-resourced CA adolescents often face. This includes considering the nuanced socioeconomic factors and potentially developing culturally tailored interventions. Finally, CA families should actively partner with their healthcare providers and local governments to participate in medical education activities.

This study identifies significant disparities between CA and aggregated AA populations and within CA populations in sociodemographic factors, healthcare access/utilization, and HPV vaccination rates. Aggregating diverse ethnic groups risks overlooking meaningful disparities, potentially hindering healthcare improvements.^55,56^ Recognizing subgroup differences is vital for data-driven, evidence-based interventions that address the unique needs of communities like CA adolescents.^57^

## Figures and Tables

**Figure 2.**
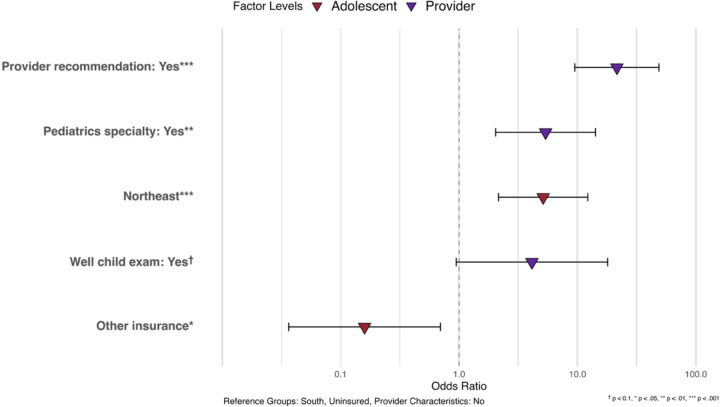
Association Between Multilevel Factors and Provider Reported HPV Vaccine Initiation in CA Adolescents, NIS-Teen 2015–2019 (N = 1,272)

**Figure 3.**
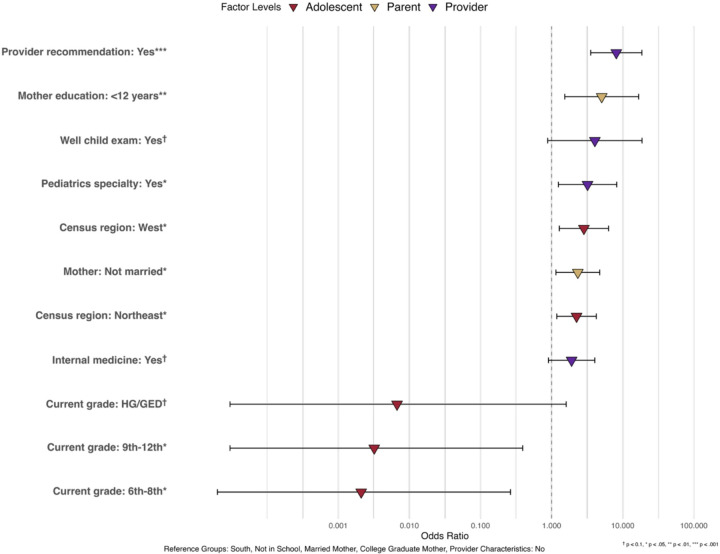
Association Between Multilevel Factors and Provider Reported HPV Vaccine Completion in CA Adolescents, NIS-Teen 2015–2019 (N = 1,272)

**Table 1:** Weighted Descriptive Statistics of Characteristics: Sociodemographic and Immigration in Adolescents and Parents, and Service Delivery in Providers/Practices (NIS-Teen 2015–2019)

Proportion or mean (95% CI)	CA (N = 1,272)	Aggregate AA (N = 5,321)
Age (mean)	14.9586 (14.7814, 15.1357)	14.99 (14.9116, 15.0684)
Sex		
Male	.3749 (.3483, .4015)	.4971 (0.4837, 0.5105)
Female	.6251 (.5985, .6517)	.5029 (0.4837, 0.5105)
Census region of residence		
Northeast	.2337 (.2104, .2570)	.2052 (0.1943, 0.2161)
Midwest	.1413 (.1222, .1604)	.1552 (0.1455, 0.1649)
South	.2068 (.1845, .2291)	.2778 (0.2658, 0.2898)
West	**.4182 (.3911, .4453)**	**.3619 (0.3490, 0.3748)**
Current grade		
6th - 8th	.2754 (.2509, .2999)	.2655 (0.2536, 0.2774)
9th to 12th	.7181 (.6934, .7428)	.7225 (0.7105, 0.7345)
HG/GED/not in school	.0065 (.0021, .0109)	.0121 (0.0092, 0.0150)
Insurance		
Private insurance only	**.7547 (.7311, .7783)**	**.6509 (0.6381, 0.6637)**
Any Medicaid	**.1503 (.1307, .1699)**	**.2383 (0.2269, 0.2497)**
Other insurance	.0626 (.0493, .0759)	.0726 (0.0656, 0.0796)
Uninsured	.0324 (.0227, .0421)	.0382 (0.0330, 0.0434)
Household income poverty level		
Above poverty >$75k	**.7063 (.6813, .7313)**	**.5929 (0.5797, 0.6061)**
Above poverty <= $75k	.2172 (.1945, .2399)	.2575 (0.2458, 0.2692)
Below poverty	.0764 (.0618, .0910)	.1496 (0.1400, 0.1592)
Nativity status		
US-born	.8724 (.8541, .8907)	.8119 (0.8014, 0.8224)
Foreign-born	.1276 (.1093, .1459)	.1881 (0.1776, 0.1986)
Marital status of mother		
Married	.8176 (.7964, .8388)	.805 (0.7944, 0.8156)
Not currently married	.1824 (.1612, .2036)	.195 (0.1844, 0.2056)
Education of mother		
< 12 years	.0505 (.0385, .0625)	.0897 (0.0820, 0.0974)
12 years	.0902 (.0745, .1059)	.1565 (0.1467, 0.1662)
>12 years, non-college grad	.1049 (.0881, .1217)	.1325 (0.1234, 0.1416)
College graduate	**.7543 (.7306, .7780)**	**.6214 (0.6084, 0.6344)**
Provider recommendation for HPV vaccine		
Yes	**.8190 (.7978, .8402)**	**.7309 (.7190, .7428)**
No	**.1810 (.1598, .2022)**	**.2691 (.2572, .2810)**
Type of facility for teens providers		
All public facilities	.0885 (.0729, .1041)	.1003 (.0922, .1084)
All private facilities	.0814 (.0664, .0964)	.0874 (.0798, .0950)
All hospital facilities	.6262 (.5996, .6528)	.6394 (.6265, .6523)
All STD/school/teen clinics or other facilities	.0487 (.0369, .0605)	.0391 (.0339, .0443)
Mixed	.1552 (.1353, .1751)	.1338 (.1247, .1429)
Specialty of provider		
Pediatrics	.7520 (.7283, .7757)	.7454 (.7337, .7571)
Family practice	.2880 (.2631, .3129)	.2928 (.2806, .3050)
General practice	.1614 (.1412, .1816)	.1371 (.1279, .1463)
Internal medicine	.2012 (.1792, .2232)	.1736 (.1634, .1838)
OB/GYN	.1503 (.1307, .1699)	.1291 (.1201, .1381)
Other	.1303 (.1118, .1488)	.1540 (.1443, .1637)
Whether provider ordered vaccines from state/local health dept		
Some or all	.8147 (.7933, .8361)	.8188 (.8085, .8292)
No	.1853 (.1639, .2067)	.1812 (.1709, .1916)
Whether provider conducted 11–12-year-old well child exam to teen		
Yes	**.9594 (.9486, .9702)**	**.9049 (.8970, .9128)**
No	**.0406 (.0298, .0514)**	**.0951 (.0872, .1030)**

Bold: *P* < .05

**Table 2:** Logistic Regression Results of Provider Reported HPV Initiation in CA Adolescents, Odds Ratio and 95% Confidence Interval, NIS-Teen 2015–2019

	Odds Ratio (95%CI), p-value
*Adolescent-level characteristics*	
Sex	
Male	Reference
Female	.9141 (.4893, 1.7079), .7780
Census region of residence	
Northeast	**5.1285 (2.1526, 12.2185), < .001** ***
Midwest	1.7274 (.7387, 4.0395), .207
South	Reference
West	1.6102 (.7115, 3.6439), .253
Insurance	
Private insurance only	1.0421 (.3033, 3.5803), .948
Any Medicaid	2.9675 (.7224, 12.1903), .131
Other insurance	**.1591 (.0364 .6951), .015** **
Uninsured	Reference
*Provider-/practice-level characteristics*	
Provider recommendation of HPV vaccine (ref: no)	**21.5026 (9.4850, 48.7472), <.001** ***
Pediatrics specialty (ref: no)	**5.374121 (2.0360, 14.1849), .001** **
Provider ordered vaccines from state/local health dept (ref: no)	.92730 (.3957, 2.1730), .862
Provider conducted 11–12-year-old well child exam to teen	4.1207 (.9448, 17.9722), .060^†^

† *p* < 0.1,

* *p* < .05,

** *p* < .01,

*** *p* < .001

**Table 3:** Logistic Regression Results of Provider Reported HPV Completion in CA Adolescents, Odds Ratio and 95% Confidence Interval (NIS-Teen 2015–2019)

*Adolescent-level characteristics*	Odds Ratio (95%CI), p-value
Sex	
Male	Reference
Female	1.29223 (.7274, 2.2957), .382
Census region of residence	
Northeast	2.2422 (1.1829, 4.2503), .013**
Midwest	1.2750 (.6362, 2.5551), .493
South	Reference
West	2.8449 (1.2816, 6.3153), .010**
Current grade	
6th - 8th	.0021 (.00002, .2643), .012**
9th to 12th	.0032 (.00003, .3920), .019**
HG/GED	.0067 (.00003, 1.6019), .073^†^
Not in school	Reference
Insurance	
Private insurance only	2.7240 (.5440, 13.64145), .223
Any Medicaid	3.1345 (6372, 15.4188), .160
Other insurance	.5326 (.0850, 3.3362), .501
Uninsured	Reference
*Parent-level characteristics*	
Marital status of mother	
Currently married	Reference
Not currently married	2.3360 (1.1489, 4.7495), .019**
Education of mother	
< 12 years	5.0600 (1.5304, 16.7295), .008**
12 years	1.9556 (.6636, 5.7634), .224
>12 years, non-college grad	.4836 (.1716, 1.3626), .169
College graduate	Reference
*Provider/practice-level characteristics*	
Provider recommendation of HPV vaccine (ref: no)	8.1190 (3.5433, 18.6036), < .001***
Pediatrics specialty (ref: no)	3.1996 (1.2447, 8.2245), .016**
Internal medicine (ref: no)	1.9101 (.9015, 4.0468), .091^†^
Provider ordered vaccines from state/local health dept (ref: no)	1.3713 (.6424, 2.9272), .414
Provider conducted 11–12-year-old well child exam to teen	4.0555 (.8806, 18.6781), .072^†^

† *p* < 0.1,

* *p* < .05,

** *p* < .01,

*** *p* < .001

## Data Availability

The NIS-Teens data are available at the CDC Website: https://www.cdc.gov/nchs/nis/data_files_teen.htm. Data requests for restricted access data can be made online through https://www.cdc.gov/rdc/application-process/accessing-restricted-data.html.

## References

[1] National Center for HIV, Viral Hepatitis, STD, and TB Prevention (U.S.). Division of STD Prevention. Sexually transmitted disease surveillance 2022 : National Overview. Published online January 30, 2024. Accessed June 9, 2025. https://stacks.cdc.gov/view/cdc/147167

[2] CDC. About Genital HPV Infection. Sexually Transmitted Infections (STIs). March 11, 2025. Accessed June 9, 2025. https://www.cdc.gov/sti/about/about-genital-hpv-infection.html

[3] CDC. Cancers Caused by HPV. Human Papillomavirus (HPV). March 3, 2025. Accessed June 9, 2025. https://www.cdc.gov/hpv/about/cancers-caused-by-hpv.html

[4] Petrosky E, Bocchini JA, Hariri S, Use of 9-valent human papillomavirus (HPV) vaccine: updated HPV vaccination recommendations of the advisory committee on immunization practices. MMWR Morb Mortal Wkly Rep. 2015;64(11):300–304. 25811679 PMC4584883

[5] Ruiz AB and NG. Key facts about Asian origin groups in the U.S. Pew Research Center. April 29, 2021. Accessed June 9, 2025. https://www.pewresearch.org/short-reads/2021/04/29/key-facts-about-asian-origin-groups-in-the-u-s/

[6] Agénor M, Pérez AE, Peitzmeier SM, Borrero S. Racial/ethnic disparities in human papillomavirus vaccination initiation and completion among U.S. women in the post-Affordable Care Act era. Ethn Health. 2020;25(3):393–407. doi:10.1080/13557858.2018.1427703 29347831

[7] Yom S, Lor M. Advancing Health Disparities Research: The Need to Include Asian American Subgroup Populations. J Racial Ethn Health Disparities. 2022;9(6):2248–2282. doi:10.1007/s40615-021-01164-8 34791615 PMC8598103

[8] Sadler GR, Ryujin L, Nguyen T, Oh G, Paik G, Kustin B. Heterogeneity within the Asian American community. Int J Equity Health. 2003;2:12. doi:10.1186/1475-9276-2-12 14697098 PMC328089

[9] Thompson CA, Gomez SL, Hastings KG, The Burden of Cancer in Asian Americans: A Report of National Mortality Trends by Asian Ethnicity. Cancer Epidemiol Biomark Prev Publ Am Assoc Cancer Res Cosponsored Am Soc Prev Oncol. 2016;25(10):1371–1382. doi:10.1158/1055-9965.EPI-16-0167 PMC5218595 27694108

[10] Bureau UC. Asian American, Native Hawaiian and Pacific Islander Heritage Month: May 2023. Census.gov. March 3, 2023. Accessed June 9, 2025. https://www.census.gov/newsroom/facts-for-features/2023/asian-american-pacific-islander.html

[11] Shing JZ, Corbin J, Kreimer AR, Human papillomavirus–associated cancer incidence by disaggregated Asian American, Native Hawaiian, and other Pacific Islander ethnicity. JNCI Cancer Spectr. 2023;7(2):pkad012. doi:10.1093/jncics/pkad012 36790075 PMC10017119

[12] Heller P. Ethnicity and cancer. 2024. Accessed June 19, 2025. https://www.ebsco.com/research-starters/health-and-medicine/ethnicity-and-cancer

[13] Nardi C, Sandhu P, Selix N. Cervical Cancer Screening Among Minorities in the United States. J Nurse Pract. 2016;12(10):675–682. doi:10.1016/j.nurpra.2016.08.036

[14] Wang Z, Jamal A, Wang R, Disparities and Trends in Routine Adult Vaccination Rates Among Disaggregated Asian American Subgroups, National Health Interview Survey 2006–2018. AJPM Focus. 2022;2(1):100044. doi:10.1016/j.focus.2022.100044 37789943 PMC10546520

[15] Office of Disease Prevention and Health Promotion. Increase the proportion of adolescents who get recommended doses of the HPV vaccine — IID-08 - Healthy People 2030. Accessed June 3, 2025. https://odphp.health.gov/healthypeople/objectives-and-data/browse-objectives/vaccination/increase-proportion-adolescents-who-get-recommended-doses-hpv-vaccine-iid-08

[16] Ma GX, Zhu L, Tan Y, A Multilevel Intervention to Increase HPV Vaccination among Asian American Adolescents. J Community Health. 2022;47(1):9–16. doi:10.1007/s10900-021-01013-z 34232452 PMC8738771

[17] Zhu L, Zhai S, Siu PT, Factors Related to Chinese Parents’ HPV Vaccinaton Intention for Children. Am J Health Behav. 2019;43(5):994–1005. doi:10.5993/AJHB.43.5.10 31439104 PMC6728078

[18] Vu M, Berg CJ, Escoffery C, A systematic review of practice-, provider-, and patient-level determinants impacting Asian-Americans’ human papillomavirus vaccine intention and uptake. Vaccine. 2020;38(41):6388–6401. doi:10.1016/j.vaccine.2020.07.059 32829979 PMC7505643

[19] Radisic G, Chapman J, Flight I, Wilson C. Factors associated with parents’ attitudes to the HPV vaccination of their adolescent sons : A systematic review. Prev Med. 2017;95:26–37. doi:10.1016/j.ypmed.2016.11.019 27932052

[20] Kessels SJM, Marshall HS, Watson M, Braunack-Mayer AJ, Reuzel R, Tooher RL. Factors associated with HPV vaccine uptake in teenage girls: a systematic review. Vaccine. 2012;30(24):3546–3556. doi:10.1016/j.vaccine.2012.03.063 22480928

[21] Walker TY. National, Regional, State, and Selected Local Area Vaccination Coverage Among Adolescents Aged 13–17 Years — United States, 2018. MMWR Morb Mortal Wkly Rep. 2019;68. doi:10.15585/mmwr.mm6833a2 PMC6705894 31437143

[22] Nguyen KH, Srivastav A, Lindley MC, Parental Vaccine Hesitancy and Association With Childhood Diphtheria, Tetanus Toxoid, and Acellular Pertussis; Measles, Mumps, and Rubella; Rotavirus; and Combined 7-Series Vaccination. Am J Prev Med. 2022;62(3):367–376. doi:10.1016/j.amepre.2021.08.015 35190101 PMC8867922

[23] Cartmell KB, Young-Pierce J, McGue S, Barriers, facilitators, and potential strategies for increasing HPV vaccination: A statewide assessment to inform action. Papillomavirus Res Amst Neth. 2018;5:21–31. doi:10.1016/j.pvr.2017.11.003 PMC5886972 29248818

[24] National Center for Immunization and Respiratory Diseases. National Immunization Surveys (NIS) - Health, United States. August 5, 2024. Accessed June 9, 2025. https://www.cdc.gov/nchs/hus/sources-definitions/nis.htm

[25] Wolter KK, Smith PJ, Khare M, Statistical Methodology of the National Immunization Survey, 2005–2014. Vital Health Stat Ser 1 Programs Collect Proced. 2017;(61):1–107. 29466229

[26] StataCorp. Stata Statistical Software: Release 16. StataCorp LLC; 2019.

[27] R Core Team. R: A Language and Environment for Statistical Computing. R Foundation for Statistical Computing; 2024. https://www.R-project.org/

[28] U.S. Department of Commerce. U.S. Census Bureau Releases Key Stats in Honor of 2023 Asian American, Native Hawaiian, and Pacific Islander Heritage Month. May 1, 2023. Accessed June 3, 2025. https://www.commerce.gov/news/blog/2023/05/us-census-bureau-releases-key-stats-honor-2023-asian-american-native-hawaiian-and

[29] Im C. Facts about Chinese in the U.S. Pew Research Center. May 1, 2025. Accessed June 3, 2025. https://www.pewresearch.org/race-and-ethnicity/fact-sheet/asian-americans-chinese-in-the-u-s/

[30] CDC. HPV Vaccination Recommendations. February 10, 2025. Accessed June 3, 2025. https://www.cdc.gov/vaccines/vpd/hpv/hcp/recommendations.html

[31] Iversen OE, Miranda MJ, Ulied A, Immunogenicity of the 9-Valent HPV Vaccine Using 2-Dose Regimens in Girls and Boys vs a 3-Dose Regimen in Women. JAMA. 2016;316(22):2411–2421. doi:10.1001/jama.2016.17615 27893068

[32] Shay LA, Street RL, Baldwin AS, Characterizing safety-net providers’ HPV vaccine recommendations to undecided parents: A pilot study. Patient Educ Couns. 2016;99(9):1452–1460. doi:10.1016/j.pec.2016.06.027 27401828 PMC5007181

[33] Vadaparampil ST, Kahn JA, Salmon D, Missed clinical opportunities: Provider recommendations for HPV vaccination for 11–12 year old girls are limited. Vaccine. 2011;29(47):8634–8641. doi:10.1016/j.vaccine.2011.09.006 21924315 PMC3200426

[34] Vadaparampil ST, Malo TL, Sutton SK, Missing the Target for Routine Human Papillomavirus Vaccination: Consistent and Strong Physician Recommendations Are Lacking for 11- to 12-Year-Old Males. Cancer Epidemiol Biomarkers Prev. 2016;25(10):1435–1446. doi:10.1158/1055-9965.EPI-15-1294 27486020 PMC5050120

[35] Gilkey MB, and McRee AL. Provider communication about HPV vaccination: A systematic review. Hum Vaccines Immunother. 2016;12(6):1454–1468. doi:10.1080/21645515.2015.1129090 PMC4964733 26838681

[36] Barnack JL, Reddy DM, Swain C. Predictors of Parents’ Willingness to Vaccinate for Human Papillomavirus and Physicians’ Intentions to Recommend the Vaccine. Womens Health Issues. 2010;20(1):28–34. doi:10.1016/j.whi.2009.08.007 20123174

[37] Bonville CA, Domachowske JB, Cibula DA, Suryadevara M. Immunization attitudes and practices among family medicine providers. Hum Vaccines Immunother. 2017;13(11):2646–2653. doi:10.1080/21645515.2017.1371380 PMC5703405 29028414

[38] Bynum SA, Staras SAS, Malo TL, Giuliano AR, Shenkman E, Vadaparampil ST. Factors Associated With Medicaid Providers’ Recommendation of the HPV Vaccine to Low-Income Adolescent Girls. J Adolesc Health. 2014;54(2):190–196. doi:10.1016/j.jadohealth.2013.08.006 24064282 PMC3946969

[39] Zhang C, Greengold J, Tackett S, Lentz C, Bennett W, McGuire M. Evaluation of online educational curriculum on HPV vaccination practices among adult primary care providers. BMC Med Educ. 2023;23:923. doi:10.1186/s12909-023-04807-y 38053126 PMC10698955

[40] Braddock AS, Bosworth KT, Ghosh P, Clinician Needs for Electronic Health Record Pediatric and Adolescent Weight Management Tools: A Mixed-Methods Study. Appl Clin Inform. 2024;15(2):368–377. doi:10.1055/a-2283-9036 38458233 PMC11078569

[41] Rodriguez V, Brignole K, Queen TL, Trogdon JG. Clinical Staff Perceptions of Pay-for-Performance Financial Incentives for HPV Vaccine Promotion. Am J Med Qual Off J Am Coll Med Qual. 2024;39(6):267–274. doi:10.1097/JMQ.0000000000000207 PMC11869125 39472771

[42] Rahman M, McGrath CJ, Berenson AB. Geographic variation in human papillomavirus vaccination uptake among 13–17 year old adolescent girls in the United States. Vaccine. 2014;32(21):2394–2398. doi:10.1016/j.vaccine.2014.02.097 24637175 PMC4062082

[43] Hirth JM, Rahman M, Smith JS, Berenson AB. Regional variations in HPV vaccination among 9–17 year old adolescent females from the BRFSS, 2008–2010. Hum Vaccines Immunother. 2015;10(12):3475–3483. doi:10.4161/21645515.2014.980202 PMC4514077 25668660

[44] Abouelella DK, Canick JE, Barnes JM, Human papillomavirus vaccine uptake among teens before and during the COVID-19 pandemic in the United States. Hum Vaccines Immunother. 18(7):2148825. doi:10.1080/21645515.2022.2148825 PMC9762824 36484115

[45] Mohammed KA. Factors Associated With Parents’ Intent to Vaccinate Adolescents for Human Papillomavirus: Findings From the 2014 National Immunization Survey–Teen. Prev Chronic Dis. 2017;14. doi:10.5888/pcd14.160314 PMC5467465 28595031

[46] Elenwo C, Batioja K, Davis T, Greiner BH, Markey C, Hartwell M. Associations of Maternal Age, Education, and Marital Status with HPV Vaccine Uptake and Hesitancy among United States Youth: A Cross-Sectional Analysis of the 2020 National Immunization Survey. J Pediatr Adolesc Gynecol. 2023;36(3):273–279. doi:10.1016/j.jpag.2023.01.213 36758721

[47] Fisher H, Audrey S, Mytton JA, Hickman M, Trotter C. Examining inequalities in the uptake of the school-based HPV vaccination programme in England: a retrospective cohort study. J Public Health. 2014;36(1):36–45. doi:10.1093/pubmed/fdt042 23620542

[48] Oza-Frank R, Kachoria R, Keim SA, Lynch CD, Klebanoff MA. Receipt and Timing of Pregnancy-Related Preventive Health Messages Vary by Message Type and Maternal Characteristics. Am J Health Promot. 2015;30(2):109–116. doi:10.4278/ajhp.131015-QUAN-524 25372233

[49] Maples JM, Zite NB, Oyedeji O, Availability of the HPV Vaccine in Regional Pharmacies and Provider Perceptions Regarding HPV Vaccination in the Pharmacy Setting. Vaccines. 2022;10(3):351. doi:10.3390/vaccines10030351 35334983 PMC8949528

[50] Osibanjo O, Benkstein K, Slater J, Shah A. The role of managed care clinical pharmacists in improving COVID-19 vaccination rates for culturally specific Medicaid populations. J Am Coll Clin Pharm. 2022;5(8):812–820. doi:10.1002/jac5.1662 35937306 PMC9347448

[51] Hu D, Taira D, Yeboah M, Castillo T. Issues Affecting Medication Use Among Asian Americans, Native Hawaiians, and Pacific Islanders: A Qualitative Study. Californian J Health Promot. 2016;14(2).

[52] Daniel CL, Lawson F, Vickers M, Enrolling a rural community pharmacy as a Vaccines for Children provider to increase HPV vaccination: a feasibility study. BMC Public Health. 2021;21(1):1266. doi:10.1186/s12889-021-11304-8 34187438 PMC8240772

[53] Olenak JL, Calpin M. Establishing a cardiovascular health and wellness program in a community pharmacy: screening for metabolic syndrome. J Am Pharm Assoc JAPhA. 2010;50(1):32–36. doi:10.1331/JAPhA.2010.08104 20097637

[54] Pingali C. Vaccination Coverage Among Adolescents Aged 13–17 Years — National Immunization Survey–Teen, United States, 2022. MMWR Morb Mortal Wkly Rep. 2023;72. doi:10.15585/mmwr.mm7234a3 PMC10468222 37616185

[55] Kauh TJ, Read JG, Scheitler AJ. The Critical Role of Racial/Ethnic Data Disaggregation for Health Equity. Popul Res Policy Rev. 2021;40(1):1–7. doi:10.1007/s11113-020-09631-6 33437108 PMC7791160

[56] Kaholokula JK, AuYoung M, Chau M, Unified in Our Diversity to Address Health Disparities Among Asian Americans, Native Hawaiians, and Pacific Islanders. Health Equity. 2022;6(1):540–545. doi:10.1089/heq.2022.0034 36186617 PMC9518805

[57] Kanaya AM, Hsing AW, Panapasa SV, Knowledge Gaps, Challenges, and Opportunities in Health and Prevention Research for Asian Americans, Native Hawaiians, and Pacific Islanders: A Report From the 2021 National Institutes of Health Workshop. Ann Intern Med. 2022;175(4):574–589. doi:10.7326/M21-3729 34978851 PMC9018596

